# An Efficient Method for Adventitious Root Induction from Stem Segments of *Brassica* Species

**DOI:** 10.3389/fpls.2016.00943

**Published:** 2016-06-29

**Authors:** Sandhya Srikanth, Tsui Wei Choong, An Yan, Jie He, Zhong Chen

**Affiliations:** Natural Sciences and Science Education, National Institute of Education, Nanyang Technological UniversitySingapore, Singapore

**Keywords:** aeroponics, *Brassica*, explants, root zone temperature, rooting, stem-segment

## Abstract

Plant propagation via *in vitro* culture is a very laborious and time-consuming process. The growth cycle of some of the crop species is slow even in the field and the consistent commercial production is hard to maintain. Enhanced methods of reduced cost, materials and labor significantly impact the research and commercial production of field crops. In our studies, stem-segment explants of *Brassica* species were found to generate adventitious roots (AR) in aeroponic systems in less than a week. As such, the efficiency of rooting from stem explants of six cultivar varieties of *Brassica* spp was tested without using any plant hormones. New roots and shoots were developed from *Brassica alboglabra* (Kai Lan), *B. oleracea* var. acephala (purple kale), *B. rapa* L. ssp. *chinensis* L (Pai Tsai, Nai Bai C, and Nai Bai T) explants after 3 to 5 days of growing under 20 ± 2°C cool root zone temperature (C-RZT) and 4 to 7 days in 30 ± 2°C ambient root zone temperature (A-RZT). At the base of cut end, anticlinal and periclinal divisions of the cambial cells resulted in secondary xylem toward pith and secondary phloem toward cortex. The continuing mitotic activity of phloem parenchyma cells led to a ring of conspicuous white callus. Root initials formed from the callus which in turn developed into ARs. However, *B. rapa* var. *nipposinica* (Mizuna) explants were only able to root in C-RZT. All rooted explants were able to develop into whole plants, with higher biomass obtained from plants that grown in C-RZT. Moreover, explants from both RZTs produced higher biomass than plants grown from seeds (control plants). Rooting efficiency was affected by RZTs and explant cuttings of donor plants. Photosynthetic CO_2_ assimilation rate (*A_sat_*) and stomatal conductance (*g_ssat_*) were significantly differentiated between plants derived from seeds and explants at both RZTs. All plants in A-RZT had highest transpiration rates.

## Introduction

Green leafy vegetables, known for their high nutritional content, are consumed by humans for good health and dietary benefits. The Brassicaceae family encompasses the Brassiceae tribe, which includes wide varieties of agriculturally and economically significant species. The genus *Brassica*, including kales, cabbages, broccoli, cauliflower, brussel sprouts, and kohlrabi, comprise of biennially herbaceous plants classified by their characteristic morphology of edible parts (Parkin et al., 2014). *B. alboglabra*, also known as Chinese kale or Kai Lan, is among the ten most marketable vegetables in Southeast Asian countries, such as Hong Kong, Thailand and China (Rakow, 2004). *B. oleracea* var. *acephala* (purple curly kale), related to the common cabbage, is a biennial temperate crop that is cultivated as an annual. Their flower buds and leaves are used as potherbs or greens (Velasco et al., 2007). Mizuna, a cultivated variety of *B. rapa* var. *nipposinica*, is a leafy vegetable commonly found in Japanese salads. Pai Tsai and Nai Bai are the cultivated varieties of *B. rapa* L. ssp. *chinensis* that is gaining popularity in Western menus where they are often steamed or stir fried (Rochfort et al., 2006). *Brassica* species play a significant role in agriculture and horticulture fields and contribute significantly to economies and population health worldwide (Zhao, 2007). Additionally, these *Brassica* species also represent an excellent system for studying numerous aspects of plant biology (Cheng et al., 2011).

Seed companies and industries face many challenges to acquire seeds from *Brassica* cultivars as it takes an especially long time to obtain. Conventional methods of hybrid seed production involve selling inbred lines for at least ten generations, while the development of homozygous plants of anther culture takes at least a year (Yang et al., 1992). Further, unlike other crops, Brassica seed production is limited to the fields with a minimum 5-year gap between seed crops and at least 2 years exclusion of any Brassica species in the same field (Ellis, 2007). Hence, a quick alternative in the commercial field would be to use vegetative propagation via stem cuttings. This minimizes seed usage while allowing for new plants to be developed within a week shorter time interval.

Vegetative propagations, such as leaves, cladode, stem or branch cuttings, are well-known methods of asexual propagations. In general, stem cutting is the most popular method of propagation for commercial plantings worldwide. However, operating costs are high as a continuous supply of fresh materials, such as peat moss, vermiculite, coir pith, root trainers, and fungicides, are required for the existing stem cutting propagation method. Rooting hormones, such as auxin, are also required for *de novo* root formation in explants. Though auxin stimulates root initiation, it also habitually leads to callus formation and expression of genes that are not necessarily related to root initiation (Welander et al., 2014). Since ARs may originate independently and directly from explant tissue rather than from callus (Fink, 1999), a more efficient rooting method with little or no callus formation would be desirable. Thus, a new method that could increase the speed of propagation whilst lower propagation costs would be an ideal alternative approach.

Soilless culture systems are useful for both research and commercial applications for food crops. An example is an aeroponic system which allows for plants to grow whilst their roots are suspended in air. As previous studies (Weathers and Giles, 1988; Zobel, 1989; Luo et al., 2012) have suggested that aeroponics is the optimum system for growing intact plants or excised roots and tissue cultures, this research explores the possibility of vegetative propagation of temperate *Brassica* species (Asian greens) in an aeroponic system within a tropical greenhouse, with the manipulation of only RZTs He et al. (2013) and He (2014) and in the absence of any hormonal applications. Therefore, this study describes a rapid and efficient rooting and whole plant regeneration methodology for six *Brassica* species. This method of root and shoot development using aeroponics can be applicable to all commercial *Brassica* cultivars. The findings of this study could be applied in the mass propagation of vegetable crops, shortening the growth cycle as seed germination and seedling development periods can be eliminated.

## Materials and Methods

### Plant Material and Growth Conditions

The six cultivars of *Brassica* spp used in the study were: Kai Lan (*B. alboglabra*), purple curly kale (*B. oleracea* var. *acephala*), Mizuna (*B. rapa* var. *nipposinica*), Pai Tsai, Nai Bai C, and Nai Bai T (*B. rapa* L. ssp. *chinensis*). The seeds were germinated at 25°C room temperature on wet tissue paper in petri plates for 3 days. Seedlings were inserted into polyurethane cubes and acclimatized at 35°C in the greenhouse for 3 days before transplanting into the aeroponic troughs at 20 ± 2°C cool root zone temperature (C-RZT) and 30 ± 2°C ambient root zone temperature (A-RZT), respectively. The roots of all plants were misted with full strength (pH 6.8, EC 2.2 mS) Netherlands Standard Composition (Douglas, 1982) nutrient solution for 1 min at 5 min intervals. All these conditions of aeroponic troughs were maintained constant throughout the experiment. Midday shoot temperatures varied between 35 to 40°C. After 45 days of transplanting, at the time of harvesting stage, few of the stem segment explants with one leaf were transplanted in their respective A- and C-RZTs to raise second generation vegetables. Another set of the seeds was germinated provided with a same nutrient solution, on the same day of explants transplantation as controls to compare the growth performance between explants and controls. Explants started rooting and established few short roots at the time of control seedling establishment in both RZTs. Hence, the establishment time period (1 week) is a prerequisite for both explants and control seeds for rooting and seed germination/seedling development, respectively. However, explants showed vigorous growth as compared to control seedlings in later stages of development.

### Plant Histology

**FIGURE 1:**
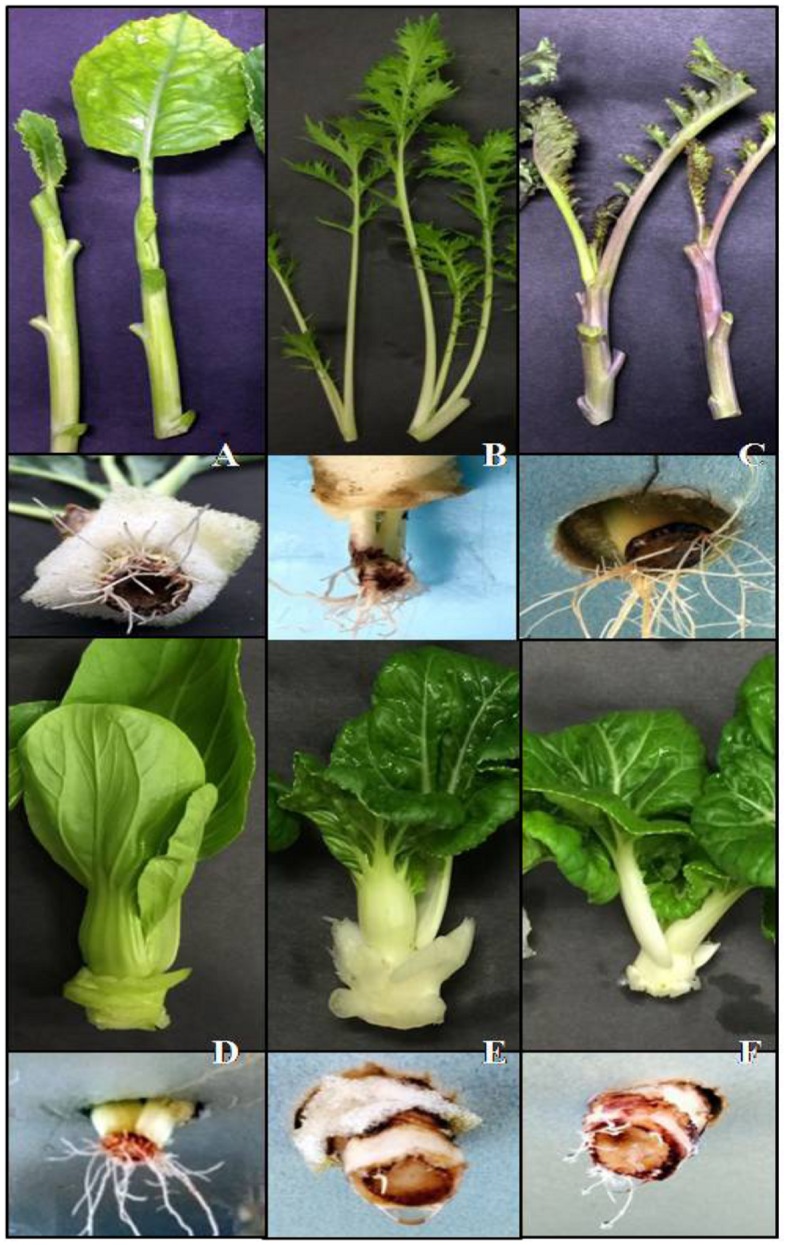
**The comparable orientation of adventitious rooting in a circular form toward the parenchymatous cortex in the cut ends of six *Brassica* sp explants. (A)** Kai Lan. **(B)** Purple curly kale. **(C)** Mizuna. **(D)** Pai Tsai. **(E)** Nai Bai C. **(F)** Nai Bai T.

Explants of all six varieties of *Brassica* species followed same rooting orientation in a circular mode toward the parenchymatous cortex region (**Figure 1**). Therefore, as an example tender and small stems of Kai Lan explant samples were collected during the callus formation and root initiation and fixed them in 10% formaldehyde for 3 days before being dehydrated through graded solutions of alcohol, cleared in xylene, and infiltrated with wax. Embedded sections were transversely and longitudinally sectioned using Leica rotary microtome at 7 μm thicknesses and transferred onto glass slides (after spreading well in a water bath at 45°C). The slides were dried on the hot plate at 45°C for 5 min. Completely dried and water free slides were dipped twice in xylene for 5 min each, twice in 100% ethanol for 5 min, once in 95% ethanol for 1 min and again once in 70% ethanol for 1min. The slides were then air dried and stained in 0.05% (w/v) toluidine blue in distilled water for 2 min, rinsed three times with deionized water to remove excess stain. The slides were then dried and mounted using Permount^TM^ mounting medium (ProSciTech, Thuringowa, Australia). Root generation from stem segments of Kai Lan was examined by using Olympus BX60 microscope.

### Measurements of Leaf Photosynthetic Pigments

Samples were collected from explants and controls after 6 weeks of their establishment in both RZTs. 0.05 g of fresh leaves of three plants of each variety were soaked in 5 ml N, *N*-dimethylformamide and left in the dark at 4°C for 48 h. Absorption at wavelengths of 480 nm, 647 nm, and 664 nm were measured using a spectrometer (UV-2550, Shimadzu, Japan) and concentrations of chlorophyll a, chlorophyll b and carotenoids were then calculated (Wellburn, 1984).

### Measurements of Photosynthetic Parameters

Newly expanded leaves of intact plants were analyzed for photosynthetic parameters such as light saturated photosynthetic CO_2_ assimilation rate (*A_sat_*), stomatal conductance (*g_ssat_*), intercellular CO_2_ concentration (*C_i_*) and transpiration rate using an open infrared gas analysis system (LI-COR), 6 weeks after the establishment of both explants and controls in C-RZT and A-RZT. The LED light source was set at a photosynthetic photon flux density of 1000 μmol m^-2^ s^-1^. Leaf chamber temperature, relative humidity, and average ambient CO_2_ concentration were 29°C, 70% and 396 ± 3 μmol mol^-1^, respectively.

### Measurements of Growth, Productivity, and Water Content

Matured explants and controls were harvested 45 days after transplant and germination, respectively, to obtain shoot and root fresh weights (FWs) and dry weights (DWs). For FW, the roots of plants were blotted with a tissue to remove excess water prior to weighing. DW was determined after drying the same plant samples at 100°C for 72 h. The means for each cultivar was determined from three plants. Morphological traits, such as leaf area, and shoot and root lengths, were also photographed and measured. Leaf area was measured using WinDIAS3 v3.2.1 (Delta-T Devices Ltd). Water content (WC) was calculated as follows: WC = [(FW-DW)/FW] × 100%.

## Results

### The Efficiency of Different *Brassica* Vegetable Explants to Generate Roots

**FIGURE 2:**
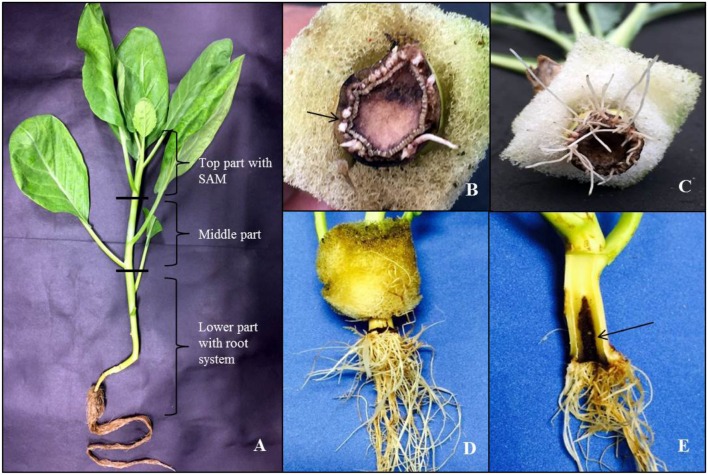
**Early events of callus formation and elongation of adventitious roots (ARs) in Kai Lan stem explants. (A)** Kai Lan plants with cutting regions and explants positions. **(B)** Arrow pointing to the callus initiation at the cut end. **(C)** Roots formed in a circular ring. **(D)** Root elongation. **(E)** Arrow showing the longitudinal section of fresh regenerating stem showing degenerating central pith region.

Stem cuttings/explants of 20 for each mode of cutting from lower, middle, and top portions (**Figure 2A**) of the donor plants were tested for the rooting ability in both RZTs to avoid highest mortality rate in explants propagation (**Table 1**). During this investigation, the highest rooting ability was found in all explants with shoot apical meristem (SAM) that grew under C-RZTwhich can be attributed to the coordination between SAM and root development in C-RZT. All explants rooted in the same level under A-RZT except Mizuna explants which died due to exposure to the highest midday light intensity up to 1000 μmolm^-2^s^-1^ and air temperature ≤40°C. KaiLan, Nai Bai C, and Nai Bai T explants from the middle and lower portions of their respective donor plants were able to root normally and developed new shoots from axillary meristems. Whereas, Pai Tsai and kale explants from middle and lower portions were unable to generate new roots and shoots and eventually died after 2–3 days of planting on both C-RZTand A-RZT.

**Table 1:** Stem segment ex-plant rooting ability in six *Brassica* species *B. alboglabra* (KaiLan), *B. oleracea* var. *acephala* (purple kale), *B. rapa* L. ssp. *Chinensis* L (Pai Tsai, Nai Bai C, and Nai Bai T).

Cultivar name	Lower stem with root system	Middle stem cuttings	Shoot apical meristem
Kai Lan	✔	✔	✔
Mizuna	✔	–	✔
Kale	✘	✘	✔
Pai Tsai	✘	✘	✔
Nai Bai C	✔	✔	✔
Nai Bai T	✔	✔	✔

### *De novo* Adventitious Root Formation in Aeroponics

**FIGURE 3:**
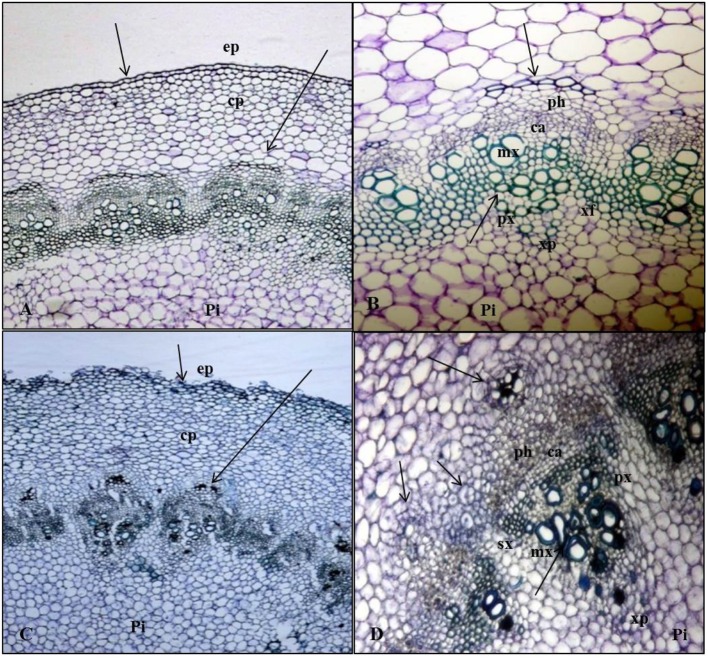
**Anatomy of AR formation from phloem parenchyma in Kai Lan stem explants of control (A,B-arrows showing normal tissues) and rooted (C,D-arrows showing the abnormal tissues and cell division) samples. (A)** Control stem transverse section showing epidermis (ep), cortex parenchyma (cp) and pith (pi) in normal tissue orientation. **(B)** Control stem showing vascular bundle (phloem (ph), cambium (ca), metaxylem (mx), protoxylem (px), xylem parenchyma (xp), and pith (pi) with aregular arrangement of cells. **(C)** Rooted stem transverse section showing irregular epidermis (ep) and cortex parenchyma (cp) with an abnormal tissue orientation. **(D)** The continuing mitotic activity of phloem parenchyma cells led to a ring of conspicuous meristematic tissue complexes evident in the cortex region of the rooted stem section. Vascular bundle showing the secondary xylem (sx) and phloem (ph) cells derived from active cambial cells (ca).

Soon after planting the stem segment explants on 20 ± 2°C and 30 ± 2°CRZTs in tropical Aeroponics, the outer layer of wounded cells died and later formed a necrotic dark layer. This layer helped to protect the cut surface from desiccation and pathogens attack. Living cells underneath this dark layer has begun divisions after 2 days of wounding and an outer layer of parenchyma cells after rapid mitotic divisions formed a circular mass of cells which later formed little white undifferentiated tissue called callus (**Figure 2B**). At the base of cut end, anticlinal and periclinal divisions of the cambial cells resulted in secondary xylem toward pith and secondary phloem toward cortex (**Figure 3**). The xylem vessels became a thick walled to ease the rapid hydraulic conductivity for regenerating explants (**Figures 3C,D**).

**FIGURE 4:**
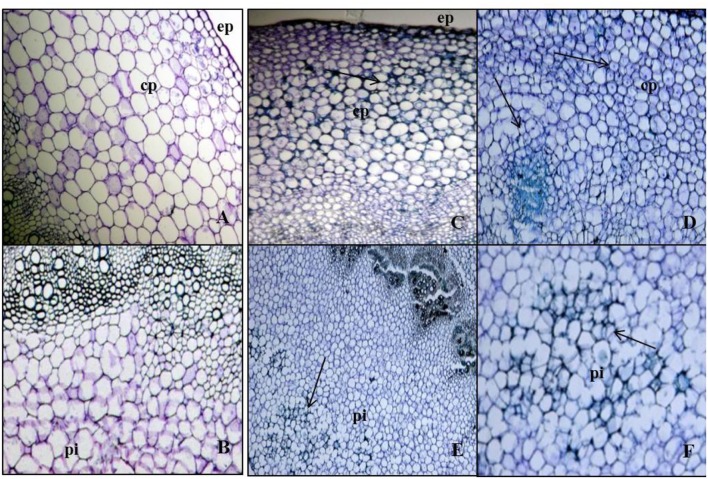
**Cell divisions in cortex and deterioration in pith occurred post root formation from the secondary phloem. (A)** Transverse section of Kai Lan control stem showing normal epidermis (ep) and cortex parenchyma (cp) regions. **(B)** Control stem showing normal pith at the center. **(C)** Transverse section of rooted stem showing active mitotic divisions in the cortex to initiate meristematic region. **(D)** Rapid mitotic divisions resulted in a circular mass of tissue in cortex parenchyma (cp). **(E)** Rooted stem showing early events of deterioration in central pith (pi). **(F)** Enlarged picture showing the cell disintegration in central pith (pi) (arrows pointing toward the cell division).

**FIGURE 5:**
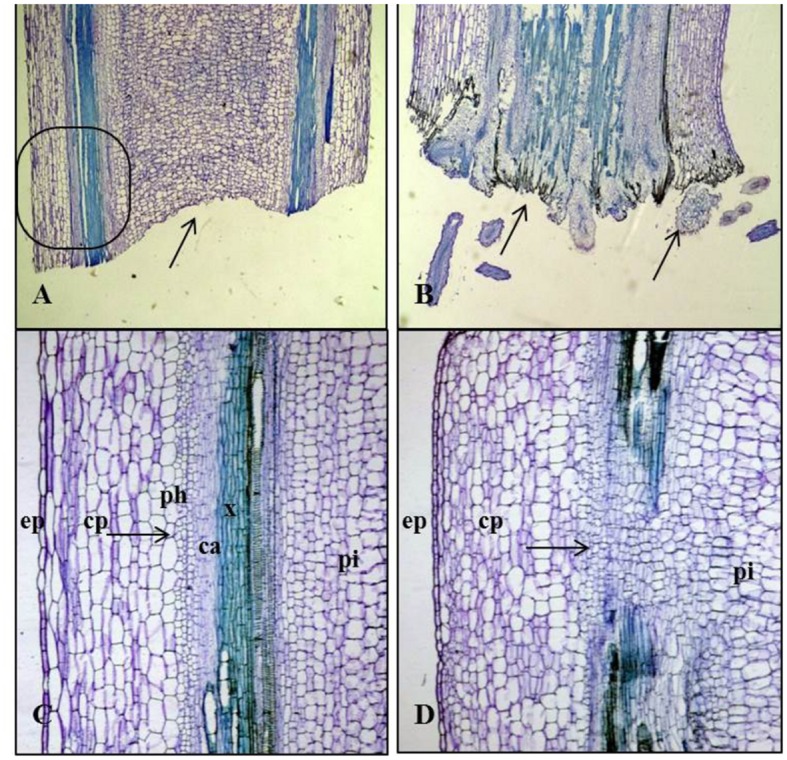
**Longitudinal sections of Kai Lan control and rooted stems showing variation in tissue orientation. (A)** Longitudinal section of the control stems (arrow indicating the blunt end without any divisions). **(B)** Longitudinal section of rooted stem showing rooting from basal wound tissue (arrow indicating the cell divisions at the cut end). **(C)** Enlarged picture of the control stem longitudinal section showing epidermis (ep), cortex parenchyma (cp), the arrow showing the regular phloem (ph), cambium (ca) xylem (x) and pith (pi). **(D)** Enlarged picture of rooted stem L.S showing epidermis (ep), cortex parenchyma (cp), pith (pi) and an arrow showing the irregular vascular bundles with rapidly dividing cells.

**FIGURE 6:**
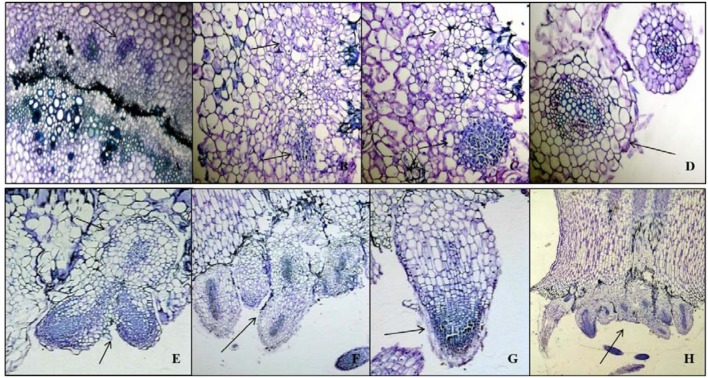
**Histological characteristics of AR development in Kai Lan stem explants. (A–D)** Transverse sections showing early events of mitotic divisions and callus initiation. **(A)** Vascular bundle showing thick-walled vascular tissues (Arrow pointing to the callus initiation from phloem parenchyma). **(B)** Arrows showing the mitotic cell divisions. **(C)** Arrows indicating the callus initials. **(D)** Arrow showing the round callus. **(E–H)** Arrows in the longitudinal sections showing root primordial initiation from callus **(E,F)**, root cap development **(G)** and AR formation from meristematic region of explant cut ends **(H)**.

The newly formed phloem parenchymatous cells after vigorous cell divisions formed root callus growth proliferations (**Figures 4**, **5**, and **6A**). Root initials formed from the callus in the vicinity of the vascular cambium and phloem ray parenchyma (**Figure 6A**), which had become meristematic by dedifferentiation. Further cell divisions occurred and the meristematic area had become more organized with the formation of a root initial (**Figures 2** and **6**). Ultimately, these root initials developed into an organized root primordia in the secondary phloem and cortex (**Figure 6A**). Later, the root primordia grew outwardly through stem tissues and formed the vascular tissue connections between the primordia and vascular tissue of stem cutting. Upon emergence from the stem segment, the ARs have already developed a root cap as well as a complete vascular connection with the originating stem (**Figures 6D–H**). Eventually, parenchymatous cells of the cortex also contributed to root formation covering the entire base with many roots and root initials (**Figures 5B** and **6H**). Visible root initials emerged from the cuttings after 3–5 days of planting in C-RZT aeroponics whereas roots were visible only after 4–7 days of planting inA-RZT. Once primordia are formed, there was a comparable time period of 5–7 days between root primordia elongation (emergence) and maximum rooting in both RZTs. Even in the C-RZT, some of the purple kale cuttings were delayed rooting even after 7 days of planting. This delay was due to variability in cuttings from different-sized stock plants but once root primordia formed, root emergence consistently occurred within a week period in both RZTs.

### Explant Establishment, Growth, and Productivity

Among six vegetable explants planted on C- and A-RZTs, Kai Lan, Nai Bai C, and Nai Bai T showed the highest survival rate as compared to other three vegetable explants such as Kale, Pai Tsai, and Mizuna. In C-RZT, Kai Lan explants exhibited 95% survival rate among all the Kai Lan cuttings planted, whereas explants in A-RZT showed 80% survival rate and 20% mortality rate. However, Nai Bai C and Nai Bai T explants showed the highest survival rate as 96 and 98% in C-RZT and 90 and 93% survival rate in A-RZT, respectively. Both Mizuna and Kale explants showed 60% survival rate in C-RZT and only Kale explants survived up to 45% in A-RZT. While the survival rate of Pai Tsai explants was 75% in C-RZT and 70% in A-RZT. In C-RZTs, control seedlings from six vegetable varieties established successfully, exhibiting up to 98% survival rate in Kai Lan, Nai Bai C, and Nai Bai T, 95% in Mizuna and Kale, whereas 96% in Pai Tsai. But in A-RZT, all six varieties showed 1 to 2% less establishment rate as compared to C-RTZ. Since all explants/cuttings used in this experiment were almost uniform in each species, the number of growth measurements such as shoot length, root length, leaf area, fresh and DWs of shoots and roots were recorded and analyzed.

**FIGURE 7:**
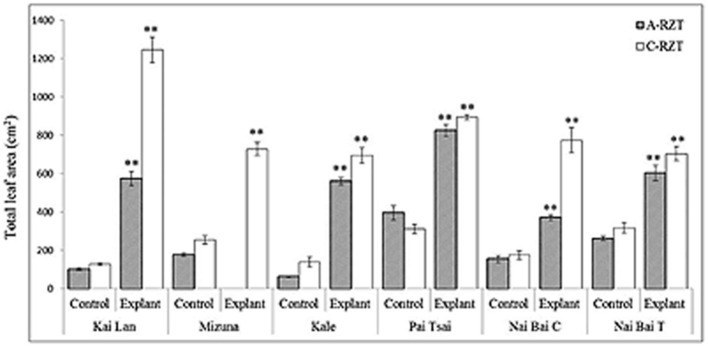
**Total leaf area of explants and controls of six cultivar varieties of *Brassica* spp. grown under C-RZT (20 ± 2°C) and A-RZT t (30 ± 2°C) in a tropical aeroponics greenhouse for 45 days.** Each bar graph is the mean of 3 measurements from three different plants (*n* ≥ 3). Vertical bars represent standard errors. Significance: ^∗∗^*p* < 0.01.

**FIGURE 8:**
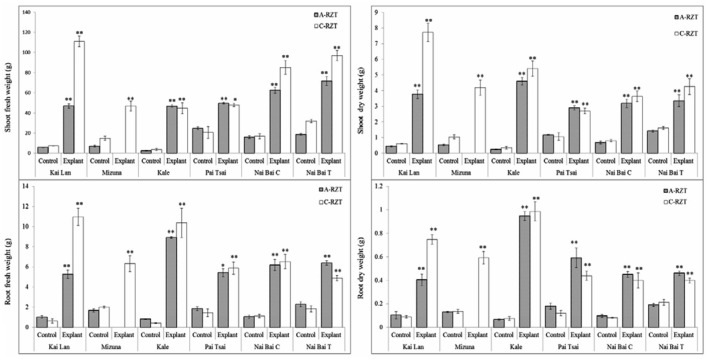
**Fresh and dry weights (DWs) of shoots and roots of explant and control plants.** Six cultivar varieties of *Brassica* spp. grown under C-RZT (20 ± 2°C) and A-RZT (30 ± 2°C) in a tropical aeroponics greenhouse for 45 days. Each bar graph is the mean of three measurements from three different plants (*n* ≥ 3). Vertical bars represent standard errors. Significance: ^∗^*p* < 0.05, ^∗∗^*p* < 0.01.

Application of different RZTs significantly affected the plant growth components, especially shoot and root lengths. Explants grown in both RZTs showed the highest biomass as compared to their controls. Nevertheless, explants of C-RZT were large stature with increased biomass compared to the explants of A-RZT. While this difference in the plant stature and biomass was also observed in the control plants of both RZTs. Moreover, all results indicating clearly that the controls were significantly smaller with very low biomass when compared with the explants in both RZTs (**Table 2**; **Figures 7** and **8**). Kai Lan explants showed almost 12.4 cm shoot length and 23.5 cm root length difference from C- to A-RZTs (**Table 2**). This larger difference in Kai Lan plant stature was also correlated with twofold decreased leaf area in A-RZT (**Figure 7**). Although there was not much difference in shoot length of Mizuna explants and controls, it was evident from the results that Mizuna explants grew well with more leaves in the C-RZT and showed almost threefold increased leaf area. Whereas kale, Pai Tsai, Nai Bai C, and Nai Bai T explants showed low to moderate differences in the plant heights and leaf area measurements, but still explants of C-RZT were significantly (^∗^*p* < 0.05, ^∗∗^*p* < 0.01) superior to the A-RZT (**Table 2**, **Figure 7**).

**Table 2:** Explants and controls showed a significant difference (^∗^*p* < 0.05, ^∗∗^*p* < 0.01; *n* = 10) in their shoot and root length under C- and A-RZTs.

Plant sample	C-RZT		A-RZT	
	Shoot length (cm)	Root length (cm)	Shoot length (cm)	Root length (cm)
Kai Lan explant	45.5 ± 1.3ˆ**	74.8 ± 3.6ˆ*	33.1 ± 2.4ˆ*	51.3 ± 1.2ˆ**
Kai Lan control	24.6 ± 0.6	51.3 ± 4.3	22.7 ± 2.6	43.6 ± 1.0
Mizuna explant	34.0 ± 2.6	83.3 ± 4.7ˆ**	–	–
Mizuna control	30.0 ± 0.5	59.5 ± 1.3	23.9 ± 1.6	28.3 ± 3.8
Kale explant	37.4 ± 2.8ˆ**	69.0 ± 4.7ˆ**	25.9 ± 4.2ˆ*	41.0 ± 1.0
Kale control	16.0 ± 1.7	38.3 ± 0.6	12.0 ± 0.5	36.8 ± 3.5
Pai Tsai explant	27.0 ± 1.1	80.0 ± 6.2ˆ*	23.3 ± 2.6	45 ± 1.5
Pai Tsai control	22.0 ± 1.6	51.3 ± 4.4	22.6 ± 1.5	38.3 ± 4.4
Nai Bai C explant	15.5 ± 0.8	61.3 ± 3.6	16.3 ± 2.7	59.6 ± 4.3ˆ*
Nai Bai C control	11.1 ± 1.6	55.0 ± 5.5	10.3 ± 0.3	29.6 ± 3.7
Nai Bai T explant	18.5 ± 1.8	69.1 ± 6.7ˆ**	14.5 ± 1.0ˆ*	51 ± 4.5
Nai Bai T control	15.1 ± 0.6	42.0 ± 3.7	10.5 ± 0.5	36.6 ± 3.6

Total fresh and DWs of shoots and roots were determined from a single harvest of three plants from each variety when explants reached harvest maturity. In correlation with the plant stature, the biomass (FW and DW) of all vegetable explants grown under cool and A- RZTs showed the highest and remarkable increase (^∗^*p* < 0.05, ^∗∗^*p* < 0.01) compared to the control plants (**Figure 8**). However, FW and DW were significantly affected by imposing different RZTs. Among all six vegetable varieties, Kai Lan explants from C-RZT showed almost twofold increased FW in concurrence with their large plant stature. However, from the data, it was evident that the DW of all vegetables decreased drastically as they possessed a high amount of water content in the root and shoots. Explants of Kai Lan shoots and roots showed 93% WC in the C-RZT whereas 92% in A-RZT. Mizuna explants possessed almost 91% WC in both roots and shoots, whereas controls showed 92% WC in both RZTs. Kale explants showed almost 90% WC in shoots and roots in both RZTs. While shoots of kale controls contained with 90.5% in C-RZT and 87% in A-RZT. But kale control roots retained 92.8% WC in A-RZT and only 84% in C-RZT. Whereas, all Pai Tsai, NaiBai C, and NaiBai T plants (both explants and controls) showed 95% WC in shoots and up to 91% WC in roots of both RZTs. From the results, it was clear that almost all plants retained more than 90% WC.

### Photosynthetic Pigments and Performance

**FIGURE 9:**
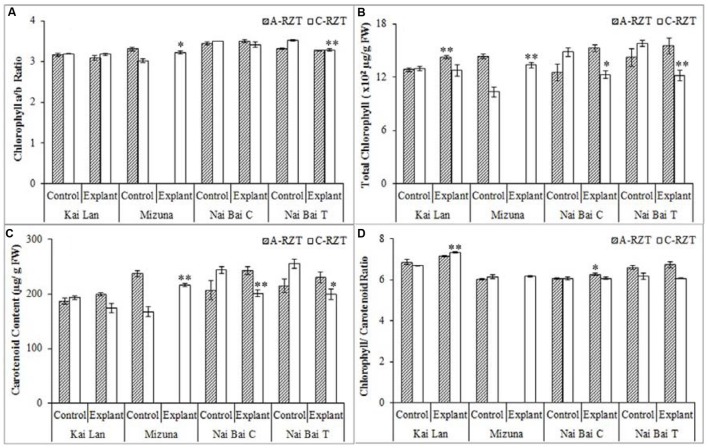
**Chlorophyll a/b ratio (A), total chlorophyll concentration (B), carotenoid concentration (C), and chlorophyll/ carotenoid ratio (D) of Kai Lan, Mizuna, Nai Bai C, and Nai Bai T grown in A-RZT and C-RZT.** Each bar graph is the mean of three measurements from three different plants (*n* ≥ 3). Vertical bars represent standard errors. Significance: ^∗^*p* < 0.05, ^∗∗^*p* < 0.01.

**FIGURE 10:**
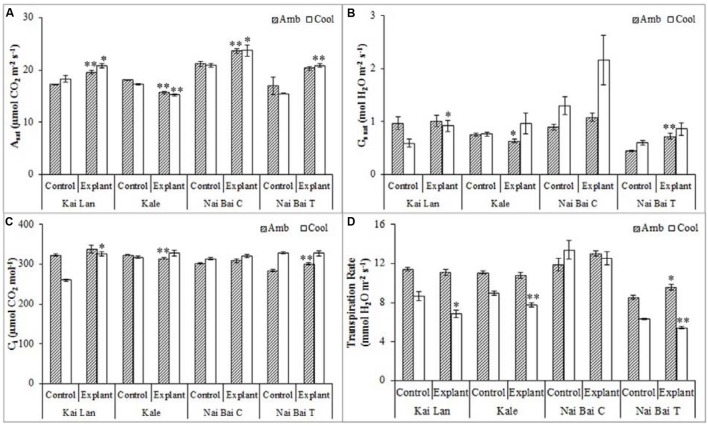
**Photosynthetic parameters such as light saturated photosynthetic CO_2_ assimilation rate (*A_sat_*) (A), stomatal conductance (*g_ssat_*) (B), intercellular CO_2_ concentration (*C_i_*) (C), and transpiration rate (D) of Kai Lan, Mizuna, Nai Bai C, and Nai Bai T grown in A-RZT and C-RZT.** Each bar graph is the mean of three measurements from three different plants (*n* ≥ 3). Vertical bars represent standard errors. Significance: ^∗^*p* < 0.05, ^∗∗^*p* < 0.01. Amb: ambient temperature; Cool: cool temperature.

Kai Lan explants had lower carotenoid content (**Figure 9C**) in C-RZT compared to A-RZT. In C-RZT, Mizuna explants had higher chlorophyll a/b ratio (**Figure 9A**), total chlorophyll (**Figure 9B**) and carotenoid content than its control plants. For Nai Bai C, there was higher total chlorophyll and carotenoid content in C-RZT for the control plants, but the reverse was observed for its explants. Such similar results were also observed in Nai Bai T, with its chlorophyll/ carotenoid ratio (**Figure 9D**) being lower for both control and explant. However, the chlorophyll/carotenoid was similar for both control and explants of Nai Bai C. *A_sat_* (**Figure 10A**) was higher for all Kai Lan plants in C-RZT, but similar between control and explants in both RZTs for the rest of the plant types. *g_ssat_* (**Figure 10B**) was generally higher for most plants in C-RZT with the exception of Kai Lan which had lower *g_ssat_*. Transpiration rate (**Figure 10D**) was generally lower in C-RZT, than A-RZT, for all plants except for Nai Bai C which had similar rates at both RZTs. Nai Bai T had higher *g_ssat_* (**Figure 10B**) and C_i_ (**Figure 10C**), though a significantly lower transpiration rate for both control and explants, at C-RZT.

## Discussion

Plant tissues have the enormous regeneration capacity, and entire plant can be developed from a single cell or small cuttings/explants (Xu and Huang, 2014). The AR formation is of great importance for vegetative propagation, but difficult to achieve in many crop species. In the present study, ARs were successfully developed from stem-segment explants of *Brassica* species on tropical aeroponics. *De novo* induction of roots from stem cuttings in plants involve the induction of meristems from adult somatic cells that are not determined to originate a meristem in normal development. Usually, AR formation is induced in stem cuttings, which experience a stimulus, such as wounding (Abarca and Díaz-Sala, 2009). Complex cellular processes involved in the AR formation are cell reorganization, induction of cell divisions, the organization of a root primordium and root development and emergence (Legué et al., 2014).

The AR formation was direct; i.e., in an organized mode without a long intervening period of callus formation. The roots grew through the cortex and often emerged out from a small round callus during the 3 to 7 day’s period after inserting the cuttings into aeroponic boards (in both RZTs). Interestingly, before root emergence, we have observed a ring of secondary vascular tissues developed from the actively dividing cambial cells at the wound site. During the rooting, this kind of tissue differentiation was observed only at the cut end and not above the wound region. Hence, it is emphasized that the wound signaling and continuous nutrient solution mist had led to the immediate auxin accumulation at the cut end which induced few small calluses and then rooting. At the base of cut end, anticlinal and periclinal divisions of the cambial cells resulted in secondary xylem toward pith and secondary phloem toward cortex. The continuing mitotic activity of secondary phloem parenchyma cells led to a ring of conspicuous white meristematic tissue complexes called ‘callus’. Root initials formed from the callus which in turn developed into ARs in the vicinity of the vascular cambium and phloem ray parenchyma. The study highlights that the hormone free cuttings can produce roots at multiple positions around the vascular tissue and so this propagation method can produce more ARs at the base of each cutting which resulted in higher survival and growth rate of explants.

In this experiment, significant results have been recorded for stem cutting propagation which would be demonstrating the possibility and success rate of vegetative propagation in tested *Brassica* samples and other vegetable species. The results are also representing the significant difference in the biomass of explants and controls. Among all, Kai Lan explants showed more remarkable FW and DW in cool RZT compared with other explants and controls. Since, explants were collected from the matured harvest stage donor plants, their well-established meristematic regions, and continuous nutrient supply through root zone area of aeroponics could be contributed for their quick recovery and root formation in a few days’ period. Once explants established the root system, they started growing vigorously and resulted in large plant stature. Meanwhile, seeds germinated on the same day of the explants planting took 2–3 days for germination and then 3 days for the establishment. But even after transplanting on the aeroponic systems, seedlings grew very slowly in the first few days until they established potential meristematic regions and root systems to support rapid growth. Eventually, seedlings of all vegetable varieties (Kai Lan, Mizuna, Kale, Pai Tsai, Nai Bai C, and Nai Bai T) started growing rapidly after one month of transplanting. While control plants reached halfway to the harvest stage, explants were grown up to harvest stage. This resulted in the high biomass content (FW and DW) of the roots and shoots of explants compared to control plants. While total leaf area was influenced by C-RZTs conditions, as there were more leaf number and specific leaf area was recorded little higher than A-RZT. However, explants recorded highest SLA compared to control plants due to changes in leaf thickness and leaf density. This simple and reliable method of explants regenerate into a whole plant, thereby rapid vegetative propagation is suitable for the vegetable varieties tested in both RZTs except Mizuna in A-RZT. But still, Mizuna showed significantly increased biomass when compared to its control plants in C-RZT. Even though Kale explants showed higher biomass content, they encountered a poor establishment problem in A-RZT. While other vegetables such as Pai Tsai, Nai Bai C, and Nai Bai T had shown greater establishment rate and yielded more in both RZTs. C-RZT also promoted the higher shoot and root lengths of Kai Lan, Mizuna, Kale, Pai Tsai, Nai Bai C, and Nai Bai T explants, suggested that plants grown in C-RZT possess a higher water and nutrition uptake capacity, which would contribute to the productivity of plants. The ratio of leaf chlorophyll a and chlorophyll b contents were not significantly different among controls and explants in both RZTs. However, total chlorophyll content is slightly higher in most of the plants in A-RZT. Nevertheless, total chlorophyll/ carotenoid ratio found to be almost similar in controls and explants of both RZT, except Nai Bai which showed a little higher ratio in A-RZT. These results demonstrating that the photosynthetic pigments contents are species specific and their performance is unique in each species. Photosynthetic parameters such as light saturated photosynthetic CO_2_ assimilation rate (*A_sat_*) is considerably higher in almost all explants at both RZTs, while stomatal conductance (*g_ssat_*) is higher in C-RZT plants. Inter cellular CO_2_ concentration (*C_i_*) is not much influenced by different RZTs and is relatively uniform among all plants. Whereas, transpiration rate is significantly higher in A-RZT plants compared to C-RZT plants except Nai Bai. Despite all variations in photosynthetic parameters, all explants were healthy and well advanced in growth in comparison to control plants. The above discussion is also demonstrating that the effect of C-RZT and A-RZT is again species-dependent.

In the present study, by using the aeroponic systems we just studied the AR formation and successful establishment of the stem segment explants of *Brassica* vegetable species without using any plant hormones, which would be useful in the vegetative propagation of leafy vegetable crops that has huge demand worldwide. Moreover, there is a need to develop different farming systems to secure the continuous vegetable production in space limited cities such as Singapore. By adapting new cultivating techniques like vegetative propagation on aeroponic farming systems, constant leafy vegetable supply is conceivable and compels the vegetable import in urban areas. There may be other instances in which aeroponic vegetative propagation can be used as an alternative to seed propagation. They include easy and rapid multiplication of selected genotypes which are generated from the conventional breeding program or induced variants from cells, tissue, or organ culture, genetic transformation, propagation of parents for hybrid seed production and speedy propagation of asexually propagated crops. Aeroponic propagation is also feasible for controlling pollination techniques such as cross pollination, self-pollination or hand pollination in the hybrid breeding program. Moreover, this method of hormone free AR formation and clonal propagation is useful for woody species that are often vegetatively propagated by stem cuttings.

## Author Contributions

ZC initiated the project. SS and TWC performed experiments. AY contributed to aeroponics plant care. SS, TWC, JH, and ZC wrote the manuscript.

## Conflict of Interest Statement

The authors declare that the research was conducted in the absence of any commercial or financial relationships that could be construed as a potential conflict of interest.
